# Nationwide registry for patients with neuroendocrine neoplasm of pancreas, gastrointestinal tract, lungs, bronchi, or thymus in Japan

**DOI:** 10.1007/s10147-022-02130-y

**Published:** 2022-02-18

**Authors:** Toshihiko Masui, Tetsuhide Ito, Izumi Komoto, Shinsuke Kojima, Yosuke Kasai, Minoru Tanabe, Kazuo Hara, Satoshi Hirano, Takuji Okusaka, Yasushi Ichikawa, Yusuke Kinugasa, Norihiro Kokudo, Atsushi Kudo, Akihiro Sakurai, Kenichi Sugihara, Hiroshi Date, Ken Haruma, Susumu Hijioka, Koichi Hirata, Hiroo Yamano, Motohiro Sakamine, Takashi Kikuchi, Masanori Fukushima, Masayuki Imamura, Shinji Uemoto

**Affiliations:** 1 Division of Hepato-Biliary-Pancreatic Surgery and Transplantation Department of Surgery, Kyoto University, Kyoto, Japan; 2 Japan Neuroendocrine Tumor Society, Kyoto, Japan; 3 School of Nursing at Fukuoka, International University of Health and Welfare, Fukuoka, Japan; 4 Hepato-Biliary-Pancreatic-Neuroendocrine-Tumor Center, Fukuoka Sanno Hospital, Fukuoka, Japan; 5 Department of Surgery, Kansai Electric Power Hospital, Osaka, Japan; 6 Division of Neuroendocrine Tumor Science, Kansai Electric Power Medical Research Institute, Osaka, Japan; 7 Translational Research Center for Medical Innovation, Kobe, Hyogo Japan; 8 Department of Surgery, Nagahama City Hospital, Nagahama, Shiga Japan; 9 Hepatobiliary and Pancreatic Surgery, Advanced Therapeutic Sciences, Medical and Dental Sciences, Graduate School of Medical and Dental Sciences, Tokyo Medical and Dental University, Tokyo, Japan; 10 Department of Gastroenterology, Aichi Cancer Center Hospital, Nagoya, Aichi Japan; 11 Department of Gastroenterological Surgery II, Graduate School of Medicine, Hokkaido University, Sapporo, Hokkaido Japan; 12 Department of Hepatobiliary and Pancreatic Oncology, National Cancer Center Hospital, Tokyo, Japan; 13 Medical Course Oncology, Graduate School of Medicine, Yokohama City University, Yokohama, Kanagawa Japan; 14 Gastrointestinal Surgery, Systemic Organ Regulation, Medical and Dental Sciences, Graduate School of Medical and Dental Sciences, Tokyo Medical and Dental University, Tokyo, Japan; 15 National Center for Global Health and Medicine, Tokyo, Japan; 16 Department of Medical Genetics and Genomics, Sapporo Medical University, Sapporo, Hokkaido Japan; 17 Specialized Surgeries, Systemic Organ Regulation, Medical and Dental Sciences, Graduate School of Medical and Dental Sciences, Tokyo Medical and Dental University, Tokyo, Japan; 18 Department of Thoracic Surgery, Kyoto University Graduate School of Medicine, Kyoto, Japan; 19 Division of Gastroenterology, Kawasaki Medical School, Okayama, Japan; 20 First Department of Surgery, Sapporo Medical University School of Medicine, Sapporo, Hokkaido Japan; 21 Department of Gastroenterology, Sapporo Medical University School of Medicine, Sapporo, Hokkaido Japan; 22 Shiga University of Medical Science, Seta Tsukinowacho, Otsu, Shiga Japan

**Keywords:** Neuroendocrine neoplasm, Prospective cohort study, Pancreas, Gastrointestinal tract, Lung, Thymus

## Abstract

**Background:**

Neuroendocrine neoplasm (NEN) is a comparatively rare tumor that has been considered indolent. Due to these characteristics, detailed epidemiological data have not been analyzed in Japan. To elucidate the present status of NEN diagnosis and treatment in Japan, we started a registry cohort study in January 2015.

**Methods:**

Patients pathologically diagnosed with NENs of the pancreas, gastrointestinal tract, lungs, bronchi, or thymus after January 2012 were enrolled in this registry after the date of ethics review committee approval in each hospital or institute. Follow-up was continued for enrolled patients.

**Results:**

During 5 years of enrollment between January 2015 and December 2019, a total of 1526 participants from 63 departments were enrolled in this registry (mean, 305.2 participants/year), covering approximately 5.8% of the annual incidence of NENs in Japan. For pancreatic NEN, 41.9% of patients had metastasis and the dominant metastatic site was the liver, at twice the rate of lymph node metastasis in the current registry. In contrast, the frequency of lymph node metastasis from gastrointestinal (GI)-NEN was similar to that of the liver. The distribution of WHO 2019-based grades varied according to the primary site. Low-to-intermediate grade (G1–G2) was dominant for duodenal, jejunal/ileal, rectal, and pancreatic NENs, whereas high grade (G3 or NEC) was dominant for esophageal, stomach, and colon NENs. For PanNENs, G3 and NEC accounted only for 1.6% and 2.9%, respectively.

**Conclusions:**

These cohort data provide crucial information for clinical research to clarify the characteristics of NENs in Japan.

## Introduction

Neuroendocrine neoplasm (NEN) was defined by the World Health Organization (WHO) in 2000 as a tumor displaying positive immunostaining for chromogranin A or synaptophysin and specific histological features. Because of the rarity of this pathology, relatively little data have been accumulated, including prevalence or incidence, especially in Japan. According to Surveillance, Epidemiology, and End Results (SEER) from the United States, the number of patients with gastroenteropancreatic NEN (GEP-NEN) has been increasing; the annual incidence rose from 1.09/100,000 population in 1973 to 5.25/100,000 population in 2004 [1]. This growth in incidence is partially due to improvements in disease recognition and diagnostic techniques [2]. In Japan, the annual incidences of pancreatic NEN (PanNEN) and GEP-NEN were 3.11/100,000 population in 2005 and 6.35/100,000 population, respectively, in 2010 according to a questionnaire-based survey [3, 4]. A recent population-based study reported that the annual incidence of GEP-NEN was 3.56/100,000 population in 2016 in Japan [5]. Although such studies provide valuable information for conducting medical practice, more detailed data are necessary to answer various clinical questions. Moreover, several differences in patient characteristics have been reported between Western countries and Japan. For example, about 30% of GEP-NEN patients in the United States registered in the SEER database have tumors in the ileum [1], compared to only around 1% of GEP-NEN patients in Japan [5]. More precise analyses using registry-based data are necessary to clarify these characteristics for GEP-NEN patients in Japan.

On the other hand, bronchopulmonary neuroendocrine tumors (BP-NETs) comprise malignant carcinomas such as large-cell neuroendocrine carcinoma (LCNEC) and small cell lung cancer (SCLC), and typical and atypical carcinoid. Such tumors form a pathologically and clinically heterogeneous group [6]. According to statistics from the Japan Surgical Society in a study of surgical cases in Japan, carcinoid was present in 198 patients (0.6%), LCNEC in 492 patients (1.4%), and SCLC in 581 patients (1.7%) among 34,228 primary lung cancer patients in 2011 [7]. LCNEC has conventionally been handled as a large-cell lung cancer with a pathology differing from that of SCLC [8]. LCNEC is a challenging tumor with a poor prognosis related to the difficulty of preoperative diagnosis.

Thymic NET is an exceedingly rare cancer, with the incidence of 0.02/100,000 population annually in the SEER report [9]. According to the Japan Surgical Society statistics from the same study of surgical cases in Japan, thymic NET was seen in only 41 patients (0.9% of 4463 thymic tumors) [7]. The clinicopathological characteristics of LCNEC and thymic NEC in Japan remain poorly understood, so detailed analysis of data for these pathological entities is needed.

With these backgrounds, we established a registry-based survey of patients with NENs of the pancreas, gastrointestinal tract, lungs, bronchi, or thymus in Japan to explore the status quo of clinical outcomes for NENs and to analyze the resulting data with the aim of contributing to future guidelines for the diagnosis and/or treatment of these entities in Japan.

## Patients and methods

This registry is a large multi-institutional prospective cohort study to clarify the actual tumor distribution and clinicopathological status in patients with NENs in Japan. Recruitment for this registry began in January 2015 and is planned to continue until November 2024. Details of the study design and protocol have been described elsewhere [10].

This study was funded by the Japan NeuroEndocrine Tumor Society (JNETS; Kyoto, Japan). The ethics review committee of Kyoto University Hospital, the Translational Research Informatics Center (TRI) of the Foundation for Biochemical Research and Innovation (Kobe, Japan) and the individual institutional review boards of all participating facilities approved this study (trial registration: UMIN000016380).

### Inclusion criteria

1. Patients histologically or pathologically diagnosed with NENs of the pancreas, gastrointestinal tract, lungs, bronchi, or thymus after 1 January 2012 who continued follow-up after the date of approval from the ethics review committee of the respective hospital or institute;
2. For GEP-NENs, patients pathologically diagnosed with NET G1/G2, NEC or mixed adenoendocrine carcinoma according to the 2010 WHO criteria [11];
3. For BP-NETs, patients pathologically diagnosed with Typical Carcinoid (TC), Atypical Carcinoid (AC), or LCNEC according to the 2004 WHO criteria [12];
4. For thymic NETs, all participants confirmed histologically or pathologically;
5. Confirmation of written informed consent from the participant (or, if the patient was < 20 years old, consent from a substitute person [an individual considered able to express the intentions and interests of the patient, such as a parental authority or legal representative of the patient]).

### Exclusion criteria

1. Patients diagnosed with SCLC,
2. Patients already registered to this study through another hospital or institution, or
3. Patients assessed as inappropriate for this research due to other reasons.

### Data collection

We collect information on baseline characteristics, clinical assessment including primary lesion, metastatic lesion, clinical TNM classification (European Neuroendocrine Society (ENETS) and Union for International Cancer Control/American Joint Committee on Cancer (UICC/AJCC)) and laboratory test results at diagnosis, and pathological findings including pathological TNM classification (ENETS and UICC/AJCC). Pathological findings are evaluated by local pathologists at each institution. Clinical stage is assessed using ENETS [13] and UICC/AJCC TNM Classification of Malignant Tumors (6th edition) [14].

Additional data on treatment and outcome surveillance have been collected since 2019 according to the revised protocol [10], providing additional baseline characteristics and outcome measurements for surgical or endoscopic resection, resection of liver metastases, adjuvant therapy, systemic therapy, locoregional therapy, and outcome surveillance including survival, progression, and recurrence.

Clinical data are obtained from medical charts by registered investigators at each participating institute and entered from a website prepared by the data center at the TRI.

### Statistical analyses

Data on outcomes are collected, including treatment information, clinical relapse and survival. Overall and disease-free survivals will be analyzed using Kaplan–Meier methods, and the prognostic impact of clinicopathological baseline factors on participant survival will be analyzed.

## Results

### Participant enrollment

**Fig. 1:**
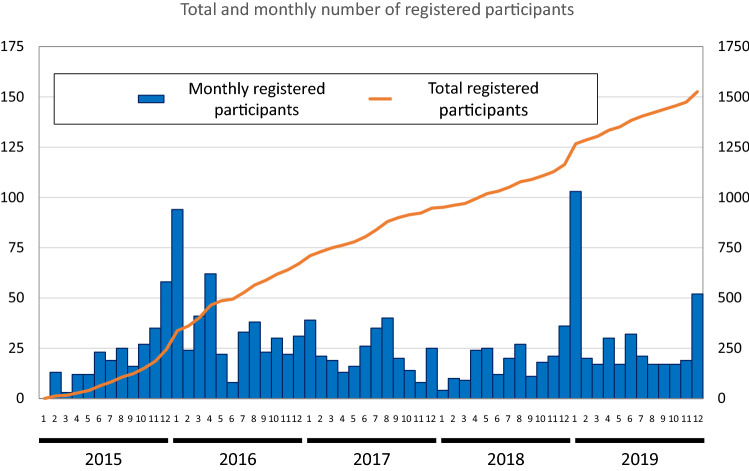
Total and monthly number of enrolled participants

During the 5 years of enrollment between January 2015 and December 2019, a total 1526 participants from 63 departments were enrolled in this registry (mean 305.2 participants/year) (Fig. 1). This registry is considered to cover approximately 5.8% of annual incidences of NENs in Japan, based on the Japanese National Cancer Registry-based incidence [5]. In 2016, a total of 248 patients were enrolled in this study. Individually, about 10.5% of pancreas NENs and 3.8% of GI-NENs, including 1.9% of rectal NENs, were registered in 2016.

The number of participants enrolled through each institution ranged from 1 to 166 in this cohort. The 34 departments that registered more than 10 cases comprised 11 departments specialized in hepatobiliary pancreatic diseases, 2 in the gastrointestinal tract, 11 in general digestive organs, 2 in pulmonary bronchial disease, and 8 in medical oncology. Baseline characteristics of participants are described in Table 1. Median age at registration was 62 years (range 0–92 years). Females accounted for 46.9% of patients. Of note, the time of registration was not always consistent with the time of initial diagnosis; for participants diagnosed before 2015, when the registry initiated, the time of registration was several years after initial diagnosis. The median time from initial diagnosis to registration was 383 days (range 0–7464 days). Median age at initial diagnosis was 60 years (range 0–88 years). Most participants had performance status of 0 (68.7%) or 1 (11.0%).

**Table 1:** General information on participants in the NET registry enrolled from January 2015 to December 2019 (*n* = 1526)

Variables	*N* (%)
General information	
Gender	
Male	810 (53.1)
Female	716 (46.9)

Age at registration	All	M^a^	F^b^
Median	62	64	60
Range	12–92	12–92	17–91
Mean	60.1	61.6	58.5
SD	13.3	12.9	13.5

Initial diagnosis	
Before 2010	58 (3.8)
2011	24 (1.6)
2012	98 (6.4)
2013	167 (10.9)
2014	223 (14.6)
2015	240 (15.7)
2016	246 (16.1)
2017	194 (12.7)
2018	146 (9.6)
2019	119 (7.8)
Unknown	11 (0.7)
Age of initial diagnosis	
Reported	1511 (99.0)
Median	60
Range	0–88
Mean	58.1
SD	13.6
Unknown date of birth	4 (0.3)
Unknown date of initial diagnosis	11 (0.7)
Time from initial diagnosis to registration	
Reported	1515 (99.3)
Median (days)	383
Range (days)	0–7464
Mean (days)	710.4
Unknown date of initial diagnosis	11 (0.7)
PS^c^	
0	1048 (68.7)
1	168 (11)
2	17 (1.1)
3	7 (0.5)
4	2 (0.1)
Not reported	284 (18.6)

^a^*n* = 809, the dates of birth of the participant is unclear and is being inquired.

^b^*n* = 713, the dates of birth of the three participants are unclear and are being inquired.

^c^Proposed by Cooperative Oncology Group (ECOG)

### Functionality and symptoms

Since this registry is ongoing, several types of data for each participant have yet to be fully inputted. Of the 1526 participants, disease characteristics were available for 1242 participants. Most participants had non-functional tumors (*n* = 1076; 86.6%), whereas 141 (11.4%) had functional tumors (Table 2). Insulinoma was the most common of the functional tumors (*n* = 68; 5.5%), followed by gastrinoma, glucagonoma, and VIPoma. Familial NENs, including multiple endocrine neoplasia type 1 (MEN1) and Von Hippel–Lindau (VHL), accounted for 4.3% of enrollments in this registry, similar to the rate reported for 2005, at 4.3% among PanNENs [3]. Hormonal or abdominal symptoms were present at the time of registration in 358 participants (28.8%).

**Table 2:** General information on participants in the NET registry enrolled from January 2015 to December 2019 (*n* = 1242)

Variables	*N* (%)
General information	
*Function/non-functional*	
Functional	141 (11.4)
Insulinoma	68 (5.5)
Gastrinoma	46 (3.7)
Glucagonoma	14 (1.1)
VIPoma	6 (0.5)
Others	9 (0.7)
Non-functional	1076 (86.6)
Unknown	25 (2.0)
*Gender*	
Familial	54 (4.3)
MEN1	43 (3.5)
VHL	10 (0.8)
TS	1 (0.1)
NF1	0 (0.0)
Others	0 (0.0)
Sporadic	1146 (92.3)
Unknown	42 (3.4)
*Symptoms*	
Symptomatic	358 (28.8)
Hypoglycemia	65 (5.2)
Diarrhea	31 (2.5)
Peptic ulcer	38 (3.1)
Diabetes mellitus	66 (5.3)
Rush	3 (0.2)
Palpitations	2 (0.2)
Abdominal pain	61 (4.9
Others	134 (10.8)
Asymptomatic	884 (71.2)

### Primary sites and metastases

Of the 1526 participants, clinical diagnosis of the primary site was available from the registry database for 1214 subjects. A breakdown of primary sites for these 1214 participants is shown in Table 3. The most common primary site was the pancreas (*n* = 702; 57.8%), followed by the rectum (*n* = 188; 15.5%) and duodenum (*n* = 97; 8.0%). A jejunal/ileal origin was seen for only 1.5%, reflecting the far lower incidence of jejunal/ileal NENs in Japan compared to Western countries. As a whole, 519 participants (42.8%) had at least one metastatic lesion (Table 3). The most common sites of metastasis were the lymph nodes and liver. More than 60% of participants with NEN of esophageal, stomach, jejunal/ileal, colon, or thymic origin had metastasis. Of note, 42 of the 188 participants (22.3%) with rectal NEN in this registry had liver metastasis.

**Table 3:** Clinical diagnosis of participants in the Net registry enrolled from January 2015 to December 2019 (*n* = 1214)

Variables		No. of cases (%)											
Clinical diagnosis		Metastasis			Metastasis organ								
Primary site	Total 1214	No 649	Not reported 46	Yes 519	Lymph node	Pancreas	Gastrointestinal tract	Liver	Thoracic organ	Bone	Brain	Skin	Others
Esophagus	16	5 (31.3)	0 (0.0)	11 (68.8)	11	0	0	5	0	0	0	0	0
Stomach	79	21 (26.6)	3 (3.8)	55 (69.6)	36	1	0	43	1	3	0	0	3
Duodenum	97	52 (53.6)	5 (5.2)	40 (41.2)	27	0	1	19	0	0	0	0	0
Jejunum/ileum	18	5 (27.8)	1 (5.6)	12 (66.7)	8	0	1	9	0	1	0	0	0
Appendix	5	4 (80.0)	0 (0.0)	1 (20.0)	1	0	0	0	0	0	0	0	0
Colon	14	2 (14.3)	1 (7.1)	11 (78.6)	8	0	1	5	0	1	0	0	2
Rectum	188	124 (66.0)	3 (1.6)	61 (32.4)	34	1	0	42	1	5	0	0	2
Pancreas	702	380 (54.1)	28 (4.0)	294 (41.9)	121	2	3	238	6	23	0	0	7
Thymus	11	2 (18.2)	2 (18.2)	7 (63.6)	3	1	0	0	0	3	0	0	2
Lung/bronchi	61	43 (70.5)	3 (4.9)	15 (24.6)	9	0	1	3	1	2	2	0	0
Others	23	11 (47.8)	0 (0.0)	12 (52.2)	7	0	0	8	0	0	0	0	0

In our current registry, a slight difference in the distribution of primary sites was seen compared to the national cancer registry-based analysis [5]; about 60% of enrollments were for PanNEN, followed by rectal NEN, but the ileum was much less frequent than reported. At least two factors may have contributed to this discrepancy; the fact that this study is still in the middle of enrollment, and the variety of departments participating in this registry. Indeed, half of the departments specialize in surgery or hepatobiliary pancreatic diseases, and such bias may affect patient enrollment. Apart from the higher enrollment of PanNEN patients, the rectum is the most common, followed by the duodenum and stomach, similar to a national cancer registry-based analysis [5]. The proportions of primary sites differed from reports from European countries, with PanNEN more frequent, and jejunal/ileal NEN was rare.

In PanNEN, 41.9% of patients had metastasis. The dominant metastatic site was the liver, at twice the rate of lymph node metastasis in the current registry. In contrast, the frequency of lymph node metastasis from GI-NEN resembled that of the liver, and differences in metastatic distribution according to primary site should be noticed. For rectal NEN, 32.4% of patients had metastasis and both lymph nodes and the liver were frequent sites of metastasis. The metastatic rate in this registry was higher compared to those in the Japanese national cancer registry and the SEER database [2, 5].

### Pathological findings

Of the 1526 participants, pathological diagnosis was available for 1041 of GEP-NENs and 71 bronchopulmonary and thymic NETs (Table 4). This registry includes data on Ki67 index and mitotic counts for participants with GEP-NENs, as components of the 2019 WHO grade classification [15]. The distribution of WHO2019-based grades varied according to the primary site, similar to a previous Japanese national cancer registry-based analysis. Low-to-intermediate grade (G1–G2) was dominant for duodenal, jejunal/ileal, rectal, and pancreatic NENs, whereas high grade (G3 or NEC) was dominant for esophageal, stomach, and colon NENs. For pancreatic NENs, G3 and NEC accounted for 1.6% and 2.9%, respectively. Higher grade was associated with more frequent incidences of metastasis in each primary site. For bronchopulmonary NETs, LCNEC was dominant (44.3%) and more than one-third of these cases showed metastatic disease. Typical and atypical carcinoids accounted for 23.0% and 14.8%, respectively.

**Table 4:** Pathological information on participants in the NET registry enrolled from January 2015 to December 2019 (*n* = 1112)

Variables* N* (%)						
Pathological information						
Percentage of WHO classification by primary sit						
				Metastasis		
				No	Yes	Not reported
Esophagus	16					
G1		0	(0.0)	0	0	0
G2		0	(0.0)	0	0	0
G3		0	(0.0)	0	0	0
NEC		10	(62.5)	3	7	0
MiNEN		1	(6.3)	0	1	0
Unknown		5	(31.3)	2	3	0
Stomach	74					
G1		9	(12.2)	9	0	0
G2		9	(12.2)	5	4	0
G3		5	(6.8)	1	4	0
NEC		34	(45.9)	3	31	0
MiNEN		6	(8.1)	0	6	0
Unknown		11	(14.9)	1	7	3
Duodenum	92					
G1		49	(53.3)	35	13	1
G2		17	(18.5)	6	10	1
G3		3	(3.3)	0	3	0
NEC		6	(6.5)	1	5	0
MiNEN		3	(3.3)	1	2	0
Unknown		14	(14.9)	8	6	0
Jejunum/ileum	16					
G1		9	(50.0)	3	5	1
G2		5	(27.8)	1	4	0
G3		0	(0.0)	0	0	0
NEC		0	(0.0)	0	0	0
MiNEN		0	(0.0)	0	0	0
Unknown		2	(22.2)	0	2	0
Appendix	5					
G1		2	(40.0)	2	0	0
G2		0	(0.0)	0	0	0
G3		0	(0.0)	0	0	0
NEC		1	(20.0)	0	1	0
MiNEN		0	(0.0)	0	0	0
Unknown		2	(40.0)	2	0	0
Colon	14					
G1		1	(7.1)	0	1	0
G2		2	(14.3)	0	2	0
G3		0	(0.0)	0	0	0
NEC		6	(42.9)	0	6	0
MiNEN		3	(21.4)	1	1	1
Unknown		2	(14.3)	1	1	0
Rectum	180					
G1		80	(44.4)	68	11	1
G2		51	(28.3)	24	27	0
G3		0	(0.0)	0	0	0
NEC		7	(3.9)	0	7	0
MiNEN		0	(0.0)	0	0	0
Unknown		42	(23.3)	29	12	1
Pancreas	623					
G1		264	(42.4)	214	44	6
G2		218	(35.0)	98	113	7
G3		10	(1.6)	2	7	1
NEC		18	(2.9)	2	16	0
MiNEN		5	(0.8)	2	3	0
Unknown		108	(17.3)	52	55	1
Others	21					
G1		3	(14.3)	3	0	0
G2		2	(9.5)	1	1	0
G3		0	(0.0)	0	0	0
NEC		4	(19.0)	1	3	0
MiNEN		5	(23.8)	1	4	0
Unknown		7	(33.3)	5	2	0
Lung/bronchi	61					
Typical carcinoid		14	(23.0)	11	1	2
Atypical carcinoid		9	(14.8)	6	3	0
LCNEC		27	(44.3)	16	10	1
Combined LCNEC		11	(18.0)	10	1	0
Others		0	(0.0)	0	0	0
Thymus	10					
Typical carcinoid		1	(10.0)	0	0	1
Atypical carcinoid		6	(60.0)	2	4	0
LCNEC		1	(10.0)	0	1	0
Combined LCNEC		0	(0.0)	0	0	0
Others		2	(20.0)	0	2	0

## Discussions

This study is the first multicenter prospective cohort study on NENs of the gastrointestinal tract, pancreas, lungs, bronchi, or thymus in Japan. Major university hospitals and high-volume institutions in Japan are participating, and around 5.8% of patients with NENs of the pancreas, gastrointestinal tract, lungs, bronchi, or thymus in Japan have been registered and their detailed data entered. Enrollment of such patients continues.

Masui et al. reported a population-based study on the epidemiology of patients with GEP-NENs from Japan. That study was based on the database of the national cancer registry and was registered by many institutions. However, registry items were comparatively few and NENs may not have been registered in that study unless considered malignant.

On the other hand, this study is enrolling all tumors diagnosed as NENs at participating institutions, regardless of whether the tumor is diagnosed as benign or malignant. This study collects more detailed information than the cancer registry, such as the actual Ki67 index, the degree of pathological differentiation, and treatment methods. The study enrolled patients diagnosed after 2012 and initially applied the 2010 WHO classification [11]. The WHO classification was updated in 2019, and NEC was classified into NET G3 and NEC according to the degree of pathological differentiation. Since both Ki67 index and the degree of pathological differentiation had been registered in the database for this study, analyses could be undertaken using the WHO 2019 classification [15]. Furthermore, the prognosis information is planned to be filled in, and to analyze the relationship of histopathology, treatment methods, with prognosis.

In the current registry, the distribution of NENs is different from the previous population-based one: there is less distribution of NENs in the lung field, and 57% of NENs are registered to be present in the pancreas and 15.5% in the rectum, which is almost the opposite of what is found in population-based studies. One reason for this is that our registry limits pulmonary NENs to typical carcinoids, atypical carcinoids, and LCNECs. These are minor components of pulmonary NENs, but they are categories that have not been fully analyzed in pulmonary NENs. Another reason is that small NENs in the colon are mainly cured by endoscopy in non-high-volume centers, and the small rate of gastrointestinal NENs may be due to differences in the distribution of registry institutions. Our current registry is being conducted to enroll patients until 2024, with around 5% of NEN patients in Japan consistently enrolled every year. With this cohort data, we are able to collect the information necessary for clinical research to clarify the characteristics of NENs in Japan and several clinical studies are ongoing. Currently, we have planned 3 clinical studies: (1) survival outcomes of non-functioning NEN with hormone-positive results from immunohistochemistry; (2) detailed analysis of somatostatin scintigraphy on high-grade NET/NEC; and (3) prognostic impact of gross typing of pancreatic NEN.

Several limitations to this study warrant consideration. First, potential exists for institutional biases in study participation. About half of the departments are specializing in surgery or hepatobiliary pancreatic diseases, and patients with a pancreatic origin or more progressive disease such as metastasis to the liver may be more likely to be enrolled. This reflects the current distribution of primary sites and the frequency of metastasis. Second, pathological data and imaging findings were reported from individual institutions and were not centrally monitored or audited, due to the constraints of manpower and cost. We plan to expand the participating departments/institutions to reduce institutional biases and intend to perform an audit to improve the accuracy of the data.

## Conclusion

In this cohort, major university hospitals and high-volume institutions in Japan are participating, and it is thought that a certain percentage of all cases with NENs of pancreas, gastrointestinal tract, lungs, bronchi, or thymus will be registered.

This cohort will be used to plan clinical studies on the pathophysiology of NENs of pancreas, gastrointestinal tract, lungs, bronchi, or thymus.

## References

[1] Yao JC, Hassan M, Phan A (2008). One hundred years after "carcinoid": epidemiology of and prognostic factors for neuroendocrine tumors in 35,825 cases in the United States. J Clin Oncol.

[2] Modlin IM, Oberg K, Chung DC (2008). Gastroenteropancreatic neuroendocrine tumours. Lancet Oncol.

[3] Ito T, Sasano H, Tanaka M (2010). Epidemiological study of gastroenteropancreatic neuroendocrine tumors in Japan. J Gastroenterol.

[4] Ito T, Igarashi H, Nakamura K (2015). Epidemiological trends of pancreatic and gastrointestinal neuroendocrine tumors in Japan: a nationwide survey analysis. J Gastroenterol.

[5] Masui T, Ito T, Komoto I (2020). Recent epidemiology of patients with gastro-entero-pancreatic neuroendocrine neoplasms (GEP-NEN) in Japan: a population-based study. BMC Cancer.

[6] Oberg K, Hellman P, Ferolla P (2012). Neuroendocrine bronchial and thymic tumors: ESMO Clinical Practice Guidelines for diagnosis, treatment and follow-up. Ann Oncol.

[7] Amano J, Kuwano H, Yokomise H (2013). Thoracic and cardiovascular surgery in Japan during 2011: Annual report by The Japanese Association for Thoracic Surgery. Gen Thorac Cardiovasc Surg.

[8] Saji H, Tsuboi M, Matsubayashi J (2010). Clinical response of large cell neuroendocrine carcinoma of the lung to perioperative adjuvant chemotherapy. Anticancer Drugs.

[9] Gaur P, Leary C, Yao JC (2010). Thymic neuroendocrine tumors: a SEER database analysis of 160 patients. Ann Surg.

[10] Masui T, Ito T, Komoto I et al (2019) Study protocol of the Japan NEN Registry: a multicenter, prospective registry of patients with pancreatic, gastrointestinal, pulmonary, bronchial, and thymic neuroendocrine neoplasm. J Clin Trials 9(6):1–7

[11] Bosman FT, Carneiro F, Hruban RH, Theise ND (2010) WHO classification of tumours of the digestive system, 4th edn, vol 3. IARC Press, Lyon, pp 10–12

[12] Travis WD, Muller-Hermelink HK, Harris CC (eds) (2004) World Health Organization Classification of tumours, pathology and genetics of tumours of the lung, pleura, tymus and heart. IARC Press, Lyon, pp 19–25

[13] Rindi G, Kloppel G, Alhman H (2006). TNM staging of foregut (neuro)endocrine tumors: a consensus proposal including a grading system. Virch Arch Int J Pathol.

[14] Frederick GP, David L, Fleming ID (2002). AJCC cancer staging manual.

[15] Nagtegaal ID, Odze RD, Klimstra D (2020). The 2019 WHO classification of tumours of the digestive system. Histopathology.

